# Transesophageal echocardiography as the final defense in impending paradoxical embolism: a case report

**DOI:** 10.1186/s13019-025-03386-x

**Published:** 2025-04-18

**Authors:** Zhuangyuan Chen, Mingjing Chen, Haibo Song, Yauwai Chan

**Affiliations:** 1 Department of Anesthesiology, The University of Hongkong-Shenzhen Hospital, Shenzhen, 518053 Guangdong People’s Republic of China; 2 Department of Epidemiology, College of Public Health and Health Professions and College of Medicine, University of Florida, Gainesville, FL USA; 3 Department of Anesthesiology, West China Hospital of Sichuan University, Chengdu, 610041 Sichuan People’s Republic of China; 4 Department of Anesthesiology, The University of Hongkong, Hongkong, 999077 People’s Republic of China

**Keywords:** Impending paradoxical embolism, Pulmonary artery embolism, Transesophageal echocardiography (TEE)

## Abstract

**Background:**

Impending paradoxical embolism (IPDE) involves venous thrombi crossing a patent foramen ovale, posing high risks of systemic embolization.

**Case presentation:**

A 64-year-old male was admitted to the operation theater due to heart failure symptoms, with the original plan of undergoing atrial mass resection and mitral valve replacement. Intra—operative transesophageal echocardiography (TEE) diagnosed the patient with IPDE and acute pulmonary embolism. And this critical diagnosis immediately altered the surgical decision-making process, which included pulmonary artery thrombectomy. The successful treatment of this case was significantly attributed to the intraoperative TEE examination, which provided essential real—time diagnostic information guiding the surgical approach. This case highlighted the importance of intra-operative TEE in management of IPDE for surgical planning.

**Conclusions:**

TEE is the final defense in IPDE. To optimize surgical outcomes and avert misdiagnosis, routine utilization of intraoperative TEE is highly recommended for similar cases.

**Supplementary Information:**

The online version contains supplementary material available at 10.1186/s13019-025-03386-x.

## Background

Paradoxical embolism occurs when venous thrombi pass through intracardiac shunts, such as patent foramen ovale (PFO), into systemic circulation, which risks systemic embolization. When a large thrombus becomes lodged within the PFO, it is classified as Impending Paradoxical Embolism (IPDE). IPDE often presents with symptoms that overlap with pulmonary embolism (PE), making it challenging to diagnose. IPDE is associated with significant mortality, with rates as high as 18.4%, primarily due to the hemodynamic instability caused by massive PE [1].

In the context of surgical management for such conditions, intraoperative transesophageal echocardiography (TEE) emerges as a crucial tool. It plays a vital role in guiding surgical decisions, predicting postoperative complications, and optimizing patient outcomes. Prior to initiating cardiopulmonary bypass (CPB), goal-directed TEE is employed to assess specific anatomical structures and detect any pathologies that could alter the surgical approach [2]. TEE not only aids in refining the surgical plan but also serves as a final safeguard to ensure patient safety by identifying incidental abnormalities.

Following anesthesia induction, systematic TEE assessment can directly detect IPDE and right heart thrombi, helping prevent missed diagnoses and providing surgeons with essential real-time information for critical decision-making. This case highlights the pivotal role of intraoperative TEE in identifying and managing IPDE. The patient provided written informed consent for the publication of his case.

Following anesthesia induction, systematic TEE assessment can directly detect IPDE and right heart thrombi. This helps prevent missed diagnoses and provides surgeons with essential real- time information for critical decision-making. This case highlights the pivotal role of intraoperative TEE in identifying and managing IPDE. The patient provided written informed consent for the publication of his case.

## Case presentation

**Fig. 1:**
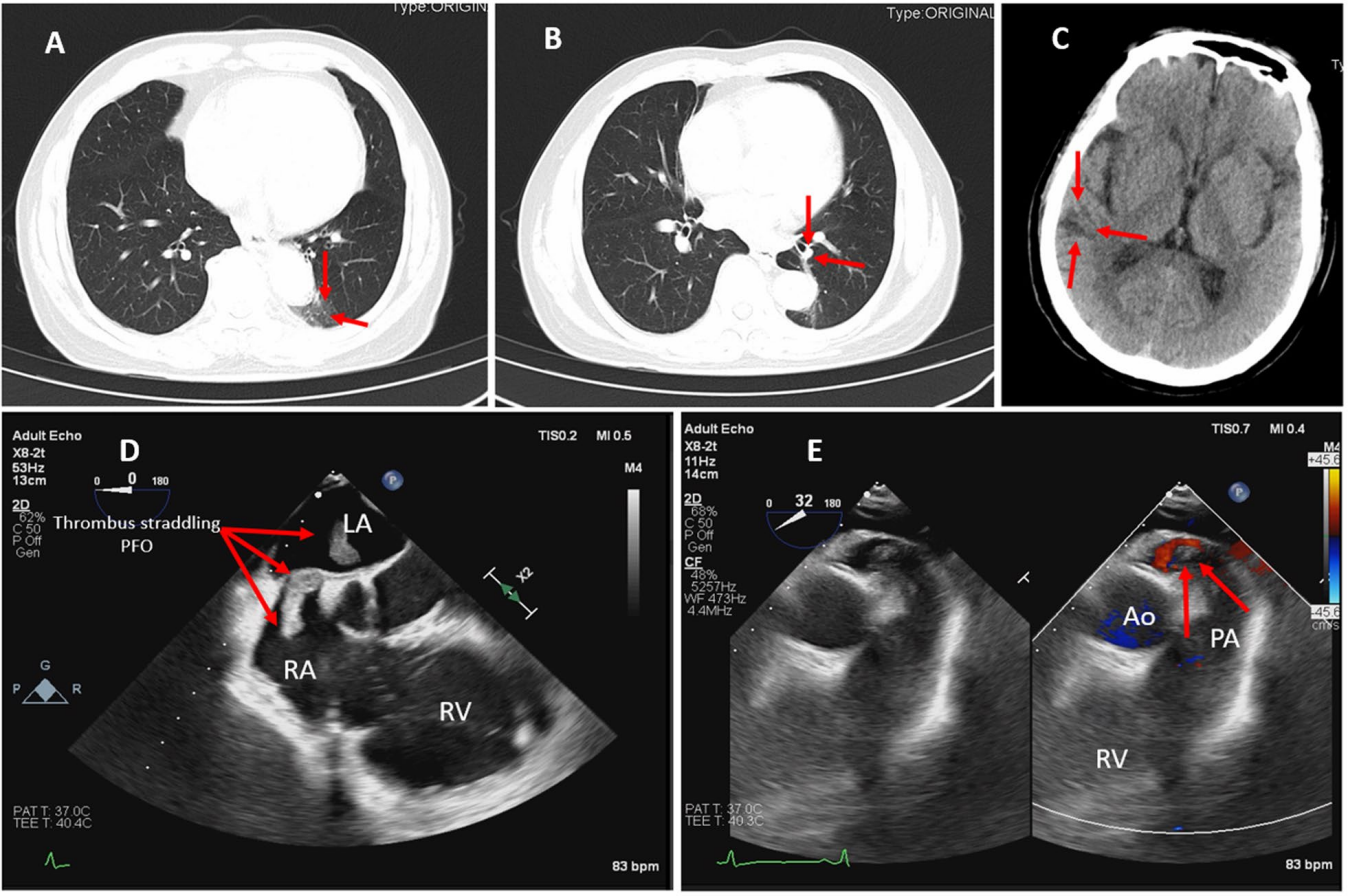
**A** Nodular shadow in the dorsal segment of the lower lobe of the left lung (red arrow). **B** Nodular shadow in the basal segment of the lower lobe of the left lung (red arrow). **C** An irregular patchy area of slightly lower density in the right temporal lobe with unclear and blurred boundaries(red arrow). **D** Modified four-chamber view: a thrombus traversing the unsealed septal PFO (red arrow), and enlargement of the right ventricle. **E**. Modified right ventricular outflow tract view: Color Doppler imaging showing a filling defect at the end of the pulmonary artery, suggestive of a thrombus (red arrow). LA: Left Atrium, RA: Right Atrium, RV: Right Ventricle. PFO: Patent Foramen Ovale. PA: Pulmonary Artery, Ao: Aorta

A 64-year-old male was admitted to the operation theater. In the weeks leading up to admission, he had been experiencing progressive dyspnea, which initially occurred during moderate- intensity physical activities such as climbing a flight of stairs. Over the past three days, the dyspnea had significantly worsened, to the extent that he was dyspneic even at rest. He also reported orthopnea, stating that he needed to sleep propped up on at least three pillows to avoid shortness of breath. Upon initial patient assessment, his vital signs were as follows: heart rate was 110 beats per minute, blood pressure was 130/80 mmHg, respiratory rate was 22 breaths per minute, and oxygen saturation on room air was 92%. An electrocardiogram (ECG) showed sinus tachycardia with non-specific ST-segment and T-wave changes. Laboratory workup revealed an elevated white blood cell count of 13 × 10⁹/L on the complete blood count (CBC). He had no significant family medical history and was previously in good health. Initial Transthoracic echocardiography (TTE) revealed right ventricular enlargement, with the right ventricular (RV) area measuring 38 cm^2^ and the right atrial area 49 cm^2^. There was an unclear echo shadow near the foramen ovale. The posterior mitral valve leaflet prolapsed, causing mild mitral regurgitation, while severe tricuspid regurgitation was present. The maximum value (Vmax) of the peak tricuspid regurgitation velocity was 3.6 m/s, resulting in a pressure gradient of 51 mmHg for the pulmonary artery systolic pressure (PASP). Additionally, during systole, elongated, band—like echogenic material was observed oscillating through the mitral valve into the left atrium, suggesting a thrombus. Computed tomography angiography (CTA) without contrast was carried out, showing irregular nodules in the basal and dorsal segments of the left lower lobe, as well as scattered small nodules throughout both lungs (Fig. 1A, B). The patient was admitted with a preliminary diagnosis of a atrial mass, mitral valve prolapse, patent foramen ovale (PFO), tricuspid valve incompetence, and a possible lung infection. The patient exhibited mild neurological symptoms. A brain CT scan was carried out, which indicated irregular hypoattenuation in the right temporal lobe, suggestive of a cerebrovascular accident, potentially suggestive of a cardio—embolic source (Fig. 1C). Considering that the nature of the cardiac mass remained uncertain, the patient refrained from receiving anticoagulant therapy before the surgery. Two days after admission, surgery was scheduled for mitral valve replacement and the repair of the patent foramen ovale (PFO). The surgical strategy entailed directly visualizing and repairing the mitral valve. Additionally, based on the specific circumstances, the surgical team was prepared to either repair or replace the tricuspid valve as necessary.

Following anesthesia induction, during the intraoperative assessment, a modified four-chamber view was obtained using a dextro-recast probe and enhanced 3D imaging. This revealed a serpentine thrombus lodged within the PFO, undulating with cardiac motion (Fig. 1D). The thrombus extended from the PFO into the left atrium during diastole, confirmed by 3D TEE (Supplementary Videos 1–3). Severe tricuspid regurgitation and significant right ventricular enlargement were also noted. Additionally, color Doppler imaging revealed a suspected thrombus within the pulmonary arteries due to a filling defect, indicating increased right heart strain and shortened pulmonary artery flow acceleration time (Fig. 1F).

**Fig. 2:**
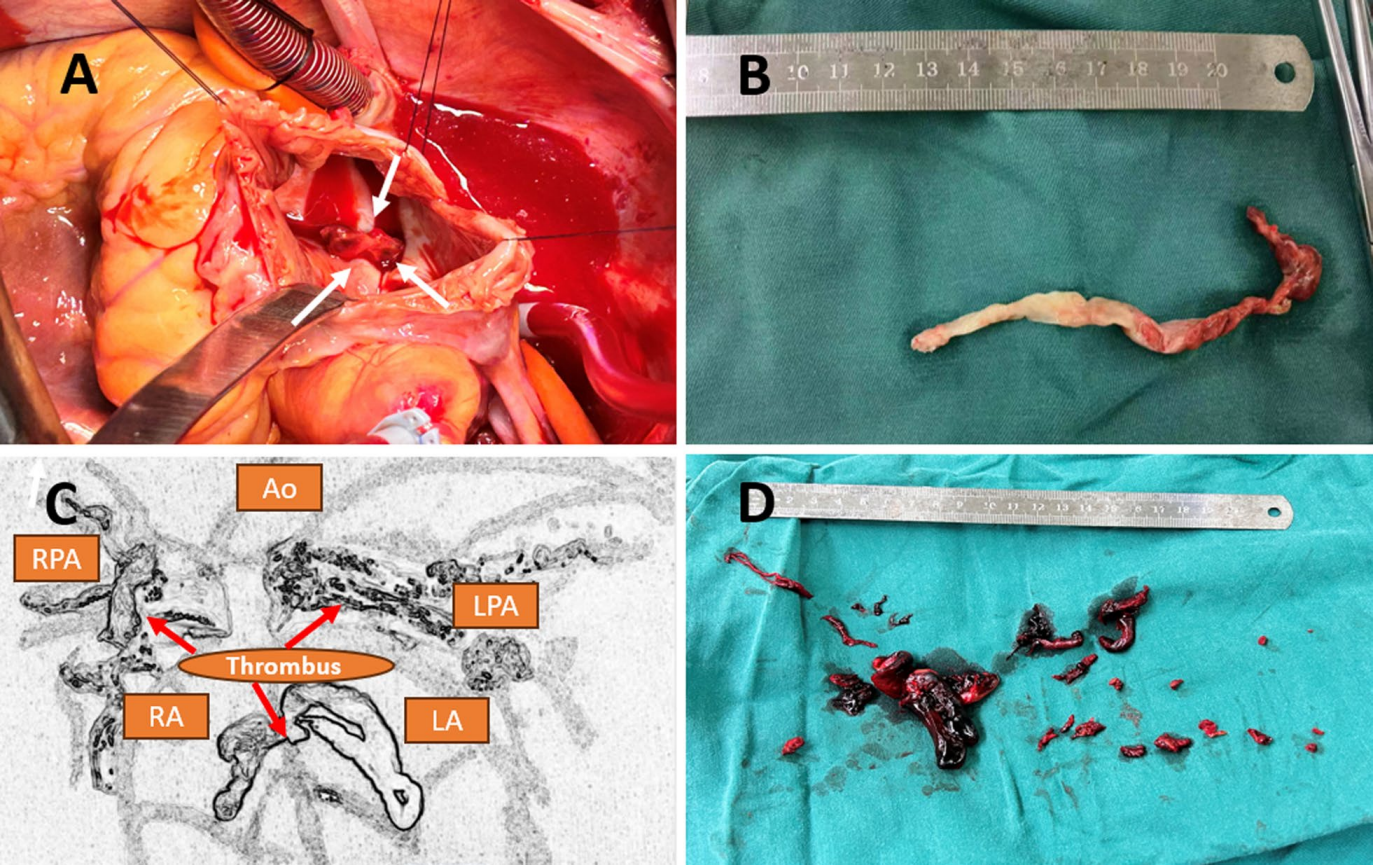
**A** In the view of the opened right atrium, a thrombus can be seen lodged at the opening of the foramen ovale (indicated by the white arrow).** B** A long thrombus was removed from the area of the foramen ovale. **C** A sketch plan of the heart vividly shows the thrombus stuck in the fossa ovalis of the interatrial septum, as well as the thrombi in the left and right pulmonary arteries. **D** A large number of thrombi were cleared from the left and right pulmonary arteries. LPA: left pulmonary artery, RPA: Right pulmonary artery

During cardiopulmonary bypass, a bar-shaped thrombus was found lodged within the PFO, measuring 3 cm × 0.3 cm towards the right atrium and 10 cm × 0.3 cm towards the left atrium (Fig. 2A–C). Additionally, large red thrombi were identified in the main branches of both pulmonary arteries and were successfully removed using suction devices (Fig. 2C, D). The annulus of the tricuspid valve was notably dilated. In response to these intraoperative findings, the surgical plan was modified to include a median sternotomy, mitral valve repair, tricuspid valve repair, excision of thrombi from both atria, and thrombectomy from both pulmonary arteries. Temporary epicardial pacing wires were also placed.

The surgery was completed successfully, and the patient was transferred to the intensive care unit (ICU) with an endotracheal tube in place. The tube was removed on the fourth postoperative day. A follow-up TTE was performed, showing an enlarged right atrium and ventricle but normal systolic function, minimal tricuspid regurgitation, a peak blood flow velocity of 1.8 m/s, an average pulmonary artery pressure of 13 mmHg, suggesting a favorable recovery. And the lower limb venous color-Doppler ultrasound examination revealed no obvious thrombus. No definitive source of the thrombus was identified during the postoperative period. The patient was discharged smoothly after 17 days of hospitalization, with significant clinical improvement and no further complications noted.

## Discussion

IPDE occurs when a thrombus becomes lodged within a PFO, creating a risk for systemic embolization. First described by Nellessen et al. [3] in 1985, IPDE is primarily reported in European Caucasians, with very few documented cases in Asia. Significant contributions to the literature on IPDE have been made by Ma [4] and Henmi [5], but without the use of 3D imaging for surgical decision-making. The successful treatment of this case was significantly attributed to the intraoperative TEE examination, which provided essential real—time diagnostic information guiding the surgical approach. This case highlighted the importance of intra-operative TEE in management of IPDE for surgical planning.

The typical presentation of IPDE involves a mass captured at the PFO, freely floating between the right and left atria. Given the high risk of arterial embolism associated with this condition, patients with concurrent PE are particularly vulnerable, with mortality rates as high as 18.4% due to the severe hemodynamic compromise caused by pulmonary artery obstruction [6]. Elevated right ventricular pressure in patients with PE can cause thrombi to migrate across the PFO, increasing the risk of systemic embolism. Clinically, IPDE can range from asymptomatic to hemodynamically unstable, complicating timely diagnosis. Thus, early detection is critical in preventing both undertreatment and missed diagnoses. Signs of right ventricular dysfunction, persistent pulmonary hypertension, an open PFO, and freely floating thrombi in the right heart all signal a heightened risk of death or recurrent thromboembolic events [7]. Furthermore, the coexistence of PE and PFO independently predicts poorer outcomes, including systemic embolism and complicated hospital courses [8].

Despite the significant risks associated with impending paradoxical embolism (IPDE), there is currently no clear consensus on the optimal treatment approach. The available treatment options encompass thrombolysis, anticoagulant therapy, interventional surgical procedures, and cardiac surgery. Both thrombolysis and anticoagulant therapy carry potential risks, such as bleeding, additional embolisms (pulmonary embolism and arterial embolism) caused by thrombus fragmentation, and hemodynamic deterioration. Moreover, interventional surgical procedures may trigger even more severe embolisms. For patients with large and mobile intracardiac thrombi, it is of utmost importance to prioritize cardiac surgery. Liu et al. [9] reported a case where the patient presented with impending paradoxical embolism, accompanied by both pulmonary embolism and ST-segment elevation myocardial infarction. Through emergency cardiac surgery and systemic anticoagulation, the patient's condition was effectively managed. Therefore, surgical intervention remains the preferred treatment option.

Intraoperative TEE has emerged as an indispensable tool for diagnosing, managing, and guiding surgical interventions in cardiac cases, particularly when preoperative transthoracic echocardiography (TTE) results are unclear. TEE provides superior visualization of intracardiac thrombi, masses, and structural obstructions, and it plays a pivotal role in modifying surgical plans when unexpected findings arise [10]. Studies by Seo et al. [11] have demonstrated that TEE has a diagnostic accuracy of 100% in detecting thrombi in PFOs, compared to an 87.3% accuracy rate for TTE.

In this case, preoperative TTE failed to detect the thrombus crossing the PFO, but intraoperative 3D TEE revealed the presence of a serpentine thrombus extending from the PFO into the left atrium. Simultaneously, TEE identified a pulmonary artery embolism that had been missed in earlier imaging. The intraoperative use of TEE was crucial in altering the surgical plan to include pulmonary artery thrombectomy, thereby preventing further embolic events. 3D TEE offers additional value by providing detailed spatial and anatomical visualization of the mass and its relationship to surrounding cardiac structures such as the atrial walls, valves, and septa. This enhanced imaging capability allows for more precise localization and characterization of the mass, helping the surgical team in planning the surgical approach. The ability of intraoperative TEE to identify and confirm structural abnormalities, while providing real-time assessments, highlights its role as a final safeguard in patient safety and decision-making. Studies have also shown that intraoperative TEE frequently leads to new findings that significantly impact surgical management. Elsherbiny et al. [12] found that routine intraoperative TEE revealed new cardiac findings in 20% of patients undergoing cardiac surgery, and in 10% of these cases, these findings led to changes in surgical plans, potentially preventing additional procedures and improving outcomes. In this case, TEE confirmed the diagnosis of IPDE through modified four-chamber views and right ventricular inflow-outflow tract (RVIOT) views. We also observed signs consistent with acute PE, including right ventricular enlargement and a shortened pulmonary artery flow acceleration time, along with the characteristic “60/60” sign-a specific indicator of PE, where right ventricular outflow tract acceleration time is less than 60 ms and the tricuspid regurgitation pressure gradient is less than 60 mmHg [13]. 

Furthermore, TEE was used to explore the pulmonary arteries using upper esophageal aortic arch short axis (UE AA SAX) and mid-esophageal ascending aortic short axis (ME Asc Aortic SAX) views. We identified thrombi in the main pulmonary arteries that were confirmed intraoperatively. Regional right ventricular dysfunction, as seen in this patient, is a common echocardiographic feature of acute PE, and the presence of McConnell’s sign—normal motion of the apex of the right ventricle with reduced movement of the remaining free wall—helped in diagnosing the severity of right ventricular dysfunction [14]. This pattern is critical in diagnosing acute PE, as it reflects the increased right ventricular load caused by acute pulmonary hypertension and its impact on ventricular function.

For managing similar cases, we employed the systematic intraoperative TEE monitoring framework described by Samuel et al. [7] and Colleen et al. [15], which provides detailed guidelines on optimizing TEE use in complex cardiac cases (Table 1). In this case, the prompt identification of IPDE and concurrent PE, combined with real-time visualization using 3D TEE, allowed for a targeted and successful surgical approach, highlighting the indispensable role of TEE in complex cardiovascular interventions.

**Table 1:** Intraoperative TEE Approach to Intracardiac IPDE and PE

Intraoperative TEE assessment	Key points of TEE focus
Basic views ME 4C/ ME LAX/ ME RVIOT/ TG Mid SAX	Is the foramen ovale patent? Are there thrombi in both atria? Is there flattening and paradoxical motion of the ventricular septum? Are there regurgitations in the tricuspid and pulmonary valves? Is there right ventricular dilation and abnormal motion? Is there impaired left ventricular diastolic function and low cardiac output?
Goal-directed views Modified 4C/RVIOT/UE AA SAX/ ME Asc Aortic SAX/ TG RV basal	Confirm the status of thrombi in the foramen ovale; presence of McConnell’s sign? Is there a suspicious thrombus in the right ventricular outflow tract, obstruction, or is the Doppler spectrum of the right ventricular outflow tract indicative of pulmonary hypertension? Presence of the 60/60 sign? Are there visible thrombi in the pulmonary arteries?
Post cardiopulmonary bypass (including above views)	Cardiac function, are preoperative abnormalities (such as pericardial/thoracic effusion, valvular regurgitation, intracardiac shunt, thrombi, valvular) repaired?

## Conclusion

This case highlights the critical role of intraoperative TEE in diagnosing and managing IPDE with PE. Intraoperative 3D TEE provided real-time visualization of the thrombus crossing the PFO and an undetected pulmonary artery embolism, enabling immediate adjustments to the surgical plan. The use of TEE in this case not only confirmed the diagnosis but also directly influenced intraoperative decisions, ensuring patient safety and successful treatment. Given its diagnostic accuracy and real-time guidance, routine use of intraoperative TEE in similar cases is recommended to optimize surgical outcomes and prevent missed diagnoses.

## Supplementary Information

Supplementary Material 1.

Supplementary Material 2.

Supplementary Material 3.

## Data Availability

No datasets were generated or analysed during the current study.

## Abbreviations

IPDE: Impending paradoxical embolism
PE: Pulmonary embolism
PFO: Patent foramen ovale
CPB: Cardiopulmonary bypass
ME RVIOT: Mid-esophageal right ventricular inflow-outflow view
ME LAX: Mid-esophageal left ventricular long axis view
TG Mid SAX: Transgastric mid short axis view
UE AA SAX: Upper esophageal aortic arch short axis view
ME Asc Aortic SAX: Mid-esophageal ascending aortic short axis view
TG RV basal: Transgastric right ventricular basal view
